# THE EFFECT OF THE JIGSAWDIO PROGRAM ON COGNITION AMONG OLDER ADULTS WITH DEMENTIA

**DOI:** 10.1093/geroni/igad104.3636

**Published:** 2023-12-21

**Authors:** Yongseop Kim, Amy Young, Jungjoo Lee, Kathleen Welsh-Bohmer, Marcia Ory, Junhyoung Kim

**Affiliations:** Indiana University, Bloomington, Indiana, United States; Jigsawdio, Bloomington, Indiana, United States; University of Southern Mississippi, Hattiesburg, Mississippi, United States; Duke University, Durham, North Carolina, United States; Texas A&M University, College Station, Texas, United States; Texas A&M University, College Station, Texas, United States

## Abstract

Puzzle activities in dementia patients have been associated with improvements in mood and cognition across a number of studies. Our research team designed and developed Jigsawdio, a digital technology that provides an innovative and interactive multisensory solution which supports memory recall in patients with dementia through the completion of personalized, audio-visual jigsaw puzzles. This device builds on the benefits derived from traditional puzzles by integrating salient audiovisual stimuli aimed at evoking nostalgia and encouraging reminiscence. The purpose of this study was to assess pre- and post-intervention effects of Jigsawdio use after six weeks among older adults with mild to moderate cognitive impairment. Using a single arm, twice weekly intervention, nine persons participated in the Jigsawdio program study. The Montreal Cognitive Assessment (MoCA) was used to measure changes in cognitive function and recall on the first and last days of the intervention. A paired t-test was used to investigate the group mean differences in cognition before and after participation in the intervention program. We found a significant group mean difference between the post-assessment and the baseline assessment MoCA scores of participants. This study presents suggestive evidence of cognitive benefits and feasibility of the technology in older adults with dementia.

